# An Intraluminal Parasitic Leiomyoma of the Sigmoid Colon and Potential Pathogenetic Mechanisms

**DOI:** 10.7759/cureus.18451

**Published:** 2021-10-03

**Authors:** Maria Dimitra Marasioni, Ermioni Tsarna, Alexios Tsochrinis, Nestor Chavez, Nikolaos Georgopapadakos

**Affiliations:** 1 Department of Obstetrics and Gynecology, General Hospital of Nikaia “Agios Panteleimon”, Piraeus, GRC

**Keywords:** leiomyoma pathogenetic mechanisms, leiomyoma pathogenesis, intestinal leiomyoma, intraluminal leiomyoma, parasitic leiomyoma

## Abstract

Uterine leiomyomas are the most common benign tumor of the female pelvis. Parasitic leiomyomas are an extremely rare entity of leiomyoma occurrence found at extrauterine sites. They are mostly diagnosed in patients with a history of gynecologic procedures and morcellators use during laparoscopic leiomyoma resection. Here we present an extraordinary case of an intraluminal leiomyoma of the sigmoid colon that was incidentally discovered during total abdominal hysterectomy and bilateral salpingo-oophorectomy, performed due to leiomyomatous uterus in a female patient with no history of previous gynecologic operations. Potential pathogenetic mechanisms that can explain the co-occurrence of leiomyomas in the uterus and the sigmoid colon are also reviewed and include genetic predisposition, the stem cell theory of leiomyomas formation, and lymphatic and vascular spread.

## Introduction

Uterine leiomyomas, also known as fibroids, are the most common benign solid tumors of the internal female genital organs. Typically arising from the uterine smooth muscle of the myometrium, leiomyomas tend to affect primarily women of reproductive age due to their hormone-dependent nature and usually involute during menopause, when ovarian steroid hormone production essentially decreases. Epidemiologically, leiomyomas seem to be more prevalent, tend to grow larger in size, and appear to be multiple in number among women of African descent compared to Caucasian and Asian women [1]. Most leiomyomas are asymptomatic and thus constitute incidental findings during imaging procedures carried out for the diagnosis of other diseases [1]. When symptomatic, leiomyomas produce symptoms depending on their size and their location throughout the uterus [1-3]. Most commonly, leiomyomas present with abdominal pain, constipation, increased urinary frequency, dysmenorrhea, and abnormal uterine bleeding [1,2]. Less frequently, leiomyomas are linked to infertility in women of reproductive age and have been found to associate with certain obstetric complications, such as fetal malpresentation, intrauterine growth restriction (IUGR), and preterm labor. The most generally accepted leiomyoma classification is the 2011 International Federation of Gynecology and Obstetrics (FIGO) classification, which outlines eight different classes of leiomyomas according to their various locations within the uterus [1]. A special category (type 8) characterizes leiomyomas with no relation to the myometrium and is used to describe those located in the cervix, the round and broad ligaments of the uterus, as well as other distant locations [1]. The latter is referred to as “parasitic” leiomyomas, a case of which will be presented hereby [1].

The aim of this case report is to describe the incidental finding of a parasitic leiomyoma inside the lumen of the sigmoid colon in a patient with leiomyomatous uterus and no history of previous laparotomy or laparoscopic procedures that would explain leiomyomatous cell translocation.

## Case presentation

A 57 years old, postmenopausal woman visited our outpatient office for gynecologic evaluation due to repeated and persistent episodes of vaginal bleeding that did not respond to treatment with progestins or estrogens, progestins, and tranexamic acid. She was gravity 0, parity 0 (G0P0), and had a known personal history of multiple uterine leiomyomas. Further complaints were persistent iron deficiency anemia, intermittent hypogastric pain, which was relieved only with non-steroid anti-inflammatory drugs (NSAIDs), and inability to achieve pregnancy during her fertile years. She had undergone several gynecologic evaluations during her reproductive years but was never offered any fertility-preserving options. Myomectomy with uterine preservation had not been considered possible, as the remaining uterine tissue would not suffice and would be unable to support a normal and healthy pregnancy. Along with the aforementioned symptoms, the patient also mentioned certain atypical symptoms. She complained of occasional bloating and constipation, for which she had visited her local general practitioner multiple times and was managed conservatively with diet modifications, only with minor improvement. At our outpatient office, the patient’s physical examination had no significant findings. A transvaginal ultrasound was performed, which revealed a large uterus with at least four leiomyomas of a maximum diameter of 3.8 cm. Ovarian imaging was not possible transvaginally due to the uterine size. The transabdominal ultrasound did not reveal any ovarian pathology. After the patient’s reevaluation and considering her present postmenopausal status, she was offered a total abdominal hysterectomy (TAH) and bilateral salpingo-oophorectomy, to which she consented.

**Figure 1:**
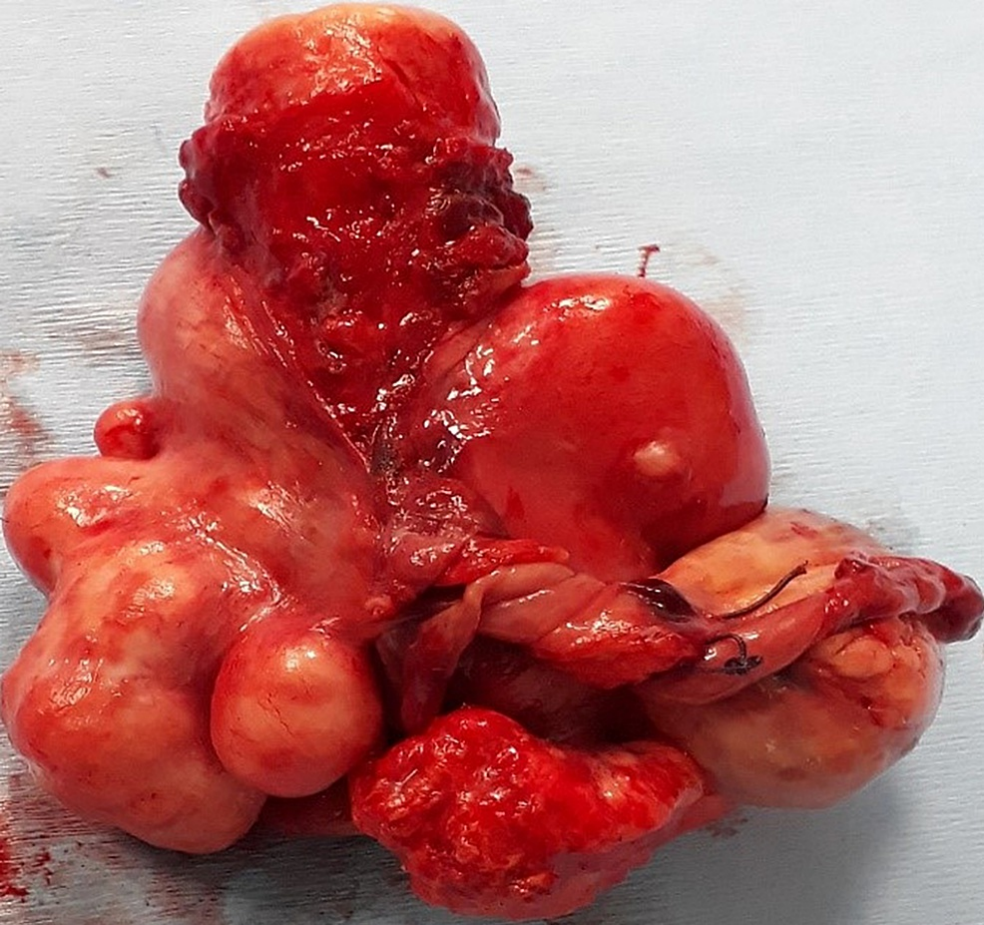
The leiomyomatous uterine specimen

**Figure 2:**
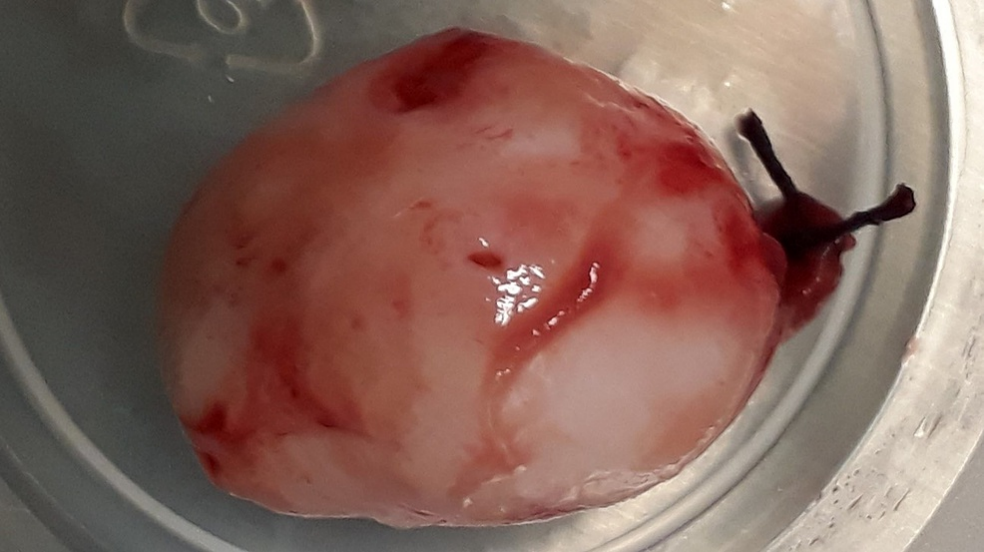
The excised intraluminal leiomyoma

Under general anesthesia, a typical TAH and salpingo-oophorectomy were performed followed by an exploration of the abdominal cavity (Figure 1). During an inspection of the Douglas pouch, a mass was observed bulging through the visceral peritoneum. In order to localize the aforementioned mass, peritoneum incision and careful tissue dissection followed, during which entry into the lumen of the sigmoid colon occurred. At this point of the operation, it became obvious that the bulging mass was located inside the lumen of the sigmoid colon rather than in the retroperitoneum and the mass was excised. Sigmoid colon resection was not regarded as necessary, as the excised mass was particularly round and smooth-edged and did not exhibit any macroscopic features compatible with malignancy. Subsequently, the defect in the sigmoid colon was closed with simple interrupted 2-0 polypropylene purse-string sutures in two layers, proper anatomy was restored, and further exploration of the abdominal cavity including spleen, liver, and the omentum was performed (Figure 2).

A histopathologic evaluation of the excised mass of the body and cervix of the uterus, the fallopian tubes, and the ovaries were ordered (Figures 1, 2). In the uterine specimen, multiple typical leiomyomas were observed, four of which were quite large, specifically of a maximum diameter of 4cm each. The excised intraluminal mass located in the sigmoid colon was sized 2.8 cm x 2.1 cm x 1.8 cm, was found to be histologically identical to the uterine leiomyomas, and eventually was reported back as typical leiomyoma. Unfortunately, the patient did not consent for immunohistochemical analysis of the specimen.

## Discussion

Parasitic leiomyomas are particularly unusual benign tumors located outside the uterus. Grouped as type 8 by FIGO, parasitic leiomyomas have no relation to the myometrium and form no attachments with the uterus. In 2009, Kimberly et al. performed a retrospective study reporting multiple cases of parasitic leiomyomas and attempted to explore their etiopathology and contributing risk factors [2]. Parasitic leiomyomas possibly originate from previous subserosal pedunculated leiomyomas that lost their attachment to the uterus, most likely due to torsion around their peduncle [2]. Subsequently, after losing their blood supply, they attempt to access the mesenteric and/or omental vessels to restore their vascularization [2]. The aforementioned was the most accepted theory before the widespread use of laparoscopy in diagnostic and therapeutic gynecological procedures. Nowadays, the most widely accepted theory of their origin is tissue translocation and abdominopelvic implantation attributed to laparoscopic gynecologic procedures and the use of morcellators, particularly during myomectomies [2].

The aforementioned theories for the etiopathogenesis of parasitic leiomyomas fail to explain rare cases of leiomyomas with unusual growth patterns that include leiomyomatosis peritonealis disseminata (LPD), intravenous leiomyomatosis, benign metastasizing leiomyomas, and retroperitoneal leiomyomas [3]. Further unusual sites of parasitic leiomyomas that cannot be explained by these theories are the vulva, the urinary bladder, the urethra, the sinonasal cavities, the orbits, the thyroid gland, the adrenal glands, the skin, and lastly intraluminally located ones in the intestines [4-6]. However interesting these rare leiomyomas may be, it remains unclear whether they share the same pathogenetic mechanisms with uterine leiomyomas or they represent a distinct pathology. The latter is supported by case reports of these rare leiomyomas in men [7].

LPD is a smooth muscle tumor that shares similar histological features to typical leiomyomas [8]. It is of unknown etiology but is ultimately believed to originate from metaplasia of peritoneal mesenchymal stem cells. It usually occurs in small nodular masses resembling peritoneal carcinomatosis [8]. This process appears to be hormonally driven since LPD has been observed in women that take oral contraceptive pills or hormone replacement therapy, are currently pregnant, or suffer from estrogen-secreting tumors [8]. Nonetheless, a growing number of recent reports suggest an association between LPD and the use of morcellators during myomectomies [8].

Intravenous leiomyomatosis is an uncommon neoplasm of smooth muscle cells diagnosed in women with symptoms suggestive of uterine leiomyomas, such as abnormal uterine bleeding and pain [9]. It usually manifests as intravascular nodules involving the pelvic and uterine veins, and rarely the inferior vena cava and the heart chambers [9]. Two main pathogenetic mechanisms have been proposed; either the tumorigenesis occurs de novo from cells within the vascular wall or through invasion of a uterine leiomyoma into the vessel itself [9]. A strong association between intravenous leiomyomatosis and chromosomal rearrangement t(12;14) resulting in HMGA2 overexpression has been made [9]. Notably, this translocation has been previously linked to uterine leiomyomas and has been described in a report of a paratesticular leiomyoma, which possibly establishes a relation between the pathogenesis of benign smooth muscle tumors of the reproductive system in both males and females [9,10].

Benign metastasizing leiomyoma is a rare entity and manifestation of extrauterine smooth muscle tumors, most commonly in the lungs, described in women with a history of current or prior leiomyomas [11]. Extrauterine benign metastasizing leiomyomas remain of unknown etiology, although similar pathogenetic mechanisms as in endometriosis have been proposed [11]. These include the phenomenon of retrograde menstruation and subsequent peritoneal seeding, iatrogenic seeding following gynecological procedures, metaplasia of the coelomic epithelium, and lastly lymphatic and hematogenous spread [11].

Retroperitoneal leiomyomas are rare benign tumors of the retroperitoneum that share the same macroscopic and microscopic features as uterine leiomyomas [7]. In the vast majority of the reported cases, the patients are perimenopausal women with a personal history of TAH for leiomyomatous uterus in approximately 40% of the cases or concomitant uterine leiomyomas [3]. Regarding their pathogenesis, it has been proposed that hormone-sensitive smooth muscle cells or embryonic remnants of the mesonephric or paramesonephric ducts, also known as Müllerian and Wolffian ducts, might be involved [7]. Similar to intravenous leiomyomatosis and benign metastasizing leiomyomas, the hematogenous spread might contribute to the development of retroperitoneal leiomyomas. Notably, retroperitoneal leiomyomas have been described in a small number of male patients [12].

Regarding intraluminal leiomyomas, they are usually incidentally discovered during routine colonoscopies in the colon or the rectum and resemble small sessile or pedunculated polyps [13]. Intraluminal leiomyomas have been also described in the small intestine. Upon removal with snare polypectomy and histologic evaluation, they manifest as small nodular round masses derived from smooth muscle tissue appearing to originate from the muscularis mucosae or the muscularis propria layer of the intestinal wall [13,14]. These leiomyomas are benign and they represent a distinct entity from the gastrointestinal stromal tumors (GISTs) [13]. They are usually diagnosed in male patients; however, cases in female patients have been also published [13]. Unfortunately, data regarding the potential concomitant presence of uterine leiomyomas in these patients are not routinely reported in the literature. Thus, the possible association between the pathogenesis of uterine and intraluminal leiomyomas remains unclear. Our case report, however, indicates that uterine and intraluminal leiomyomas might be causally associated. We, further propose that genetics or stem cell-driven neoplastic proliferation may mediate this association.

Recently, studies on animal and human models have led to the identification of myometrial stem cells (MyoSCs) [15,16]. MyoSCs, possess multipotent cell properties and are believed to mediate the pregnancy-associated uterine expansion, which is characterized by hypertrophy/hyperplasia and exhibits extensive tissue plasticity and regeneration [16]. Similarly, the post-delivery involution of the uterus to its previous weight and volume, which is characterized by myometrial cell apoptosis, is controlled by MyoSCs [15]. While studying uterine tissue expansion and involution on a molecular level, certain potential MyoSC surface markers have emerged [15]. Four main surface markers have been identified; namely the CD49f, CD34, and the combined STRO-I/CD44 that express strong features of tumor-initiating cells (TICs) [15]. Under hypoxic conditions, cells possessing these markers have been shown to drive cell colony formation and tissue regeneration, in in vitro and in vivo models respectively [15]. Animal models have indicated the presence of a stem cell niche, typically located in a distinct anatomic location [15]. Extrinsic factors, such as tissue hypoxia, act on the stem cell niche, which eventually mediates MyoSCs proliferation and therefore myometrial tissue homeostasis [15]. Uterine leiomyoma formation has been studied in Eker rat murine models, in which the hypoxic niche located in the cervix of these animals appears to be the first site of leiomyoma formation [15]. The state of tissue hypoxia, possibly during myometrial contraction and vasoconstriction, dysregulates MyoSCs to TICs and ultimately mediates uterine tumor formation by activating estrogen signaling pathways [15]. The aforementioned model is yet to be studied in humans [15]. In animal models, locally produced factors mediate cellular communication in the MyoSC niche via paracrine and autocrine mechanisms [15]. The factors mostly implicated in the cellular events acting on the MyoSC are epidermal growth factor (EGF), platelet-derived growth factor, transforming growth factor-beta (TGF-β), myostatin, and activin [15]. The aforementioned activate a number of different pathways that together with ovarian hormones contribute to leiomyoma formation [15]. In contrast to uterine leiomyomas, the potential role of stem cells in the pathogenesis of smooth muscle tumors of the gastrointestinal tract is markedly understudied. Due to their rarity, benign tumors of the gastrointestinal tract originating from smooth muscle cells are not as routinely studied as are the neoplasms arising from the epithelial cells. Up to date, it remains unknown whether intestinal stem cells can give rise to non-epithelial cells of the intestines; even, in that case, the conditions that stimulate such a process and the underlying molecular mechanisms remain unknown. Thus, the potential role of stem cells in the formation of intraluminal leiomyomas of the colon is not explored in the literature, and, as follows, this case report is merely suggestive of a possible common stem cell-based pathogenetic process that is yet to be studied.

With regards to genetics, it is well established that uterine leiomyomas are more prevalent among women of African descent, regardless of the place of residence [1]. This indicates that uterine leiomyomas have a genetic background, possibly along with environmental risks factors. However, since intraluminal leiomyomas of the sigmoid colon are very rare entities, relevant epidemiologic data are lacking. Thus, genetic predisposition cannot be implicated based on epidemiological data. Considering data arising from DNA sequencing, four main genes have been involved in uterine leiomyomas formation, namely MED12 gene mutations, overexpression of the HMGA2 gene, inactivation of fumarate hydratase gene, and deletions of collagen alpha (COL4A6-COL4A5) gene [15]. Only MED12 and HMGA2 mutations seem to affect stem cell function and proliferation that potentially drive leiomyoma formation by generating TICs, thus supporting the stem cell theory [15]. MED12 mutations not only give rise to uterine tumors, both benign and malignant, but have been also associated with extrauterine smooth muscle tumors and other miscellaneous ones rarely arising in the colon, breast, and prostate [16]. Somatic MED12 mutations on exon 2 drive tumorigenesis in extrauterine smooth muscle tissue locations of Mullerian origin, specifically in the pelvis and the retroperitoneum of female patients, and have been also described in intravenous leiomyomatosis [17]. Ravegnini et al. managed to demonstrate exon 2 MED12 gene mutations in ovarian, kidney, and retroperitoneal leiomyomas [18]. However, no exon 2 somatic mutations of the MED12 gene have been identified in extrauterine extrapelvic leiomyomas [17]. Considering the large intestine, the genetic background of colonic leiomyomas remains unclear [19]. Nevertheless, deletions of the COL4A5/COL4A6 on the X-chromosome have been linked to certain cases of esophageal leiomyomas found predominantly in men [19,20].

## Conclusions

Uterine leiomyomas are common smooth muscle tumors of the female reproductive tract. In contrast, intraluminal parasitic leiomyomas of the colon, a case of which we have presented here, are markedly uncommon. The concurrence of the aforementioned entities indicates that common pathogenetic mechanisms might be shared. Genetic predisposition, the stem cell theory of leiomyomas formation, and lymphatic and vascular spread potentially explain the rare occurrence of an intraluminal parasitic leiomyoma of the sigmoid colon.
